# Tualang Honey Improves Human Corneal Epithelial Progenitor Cell Migration and Cellular Resistance to Oxidative Stress *In Vitro*

**DOI:** 10.1371/journal.pone.0096800

**Published:** 2014-05-06

**Authors:** Jun Jie Tan, Siti Maisura Azmi, Yoke Keong Yong, Hong Leong Cheah, Vuanghao Lim, Doblin Sandai, Bakiah Shaharuddin

**Affiliations:** 1 Advanced Medical and Dental Institute, Universiti Sains Malaysia, Kepala Batas, Penang, Malaysia; 2 Department of Human Anatomy, Faculty of Medicine and Health Sciences, Serdang, Selangor Darul Ehsan, Malaysia; Georgetown University, United States of America

## Abstract

Stem cells with enhanced resistance to oxidative stress after *in vitro* expansion have been shown to have improved engraftment and regenerative capacities. Such cells can be generated by preconditioning them with exposure to an antioxidant. In this study we evaluated the effects of Tualang honey (TH), an antioxidant-containing honey, on human corneal epithelial progenitor (HCEP) cells in culture. Cytotoxicity, gene expression, migration, and cellular resistance to oxidative stress were evaluated. Immunofluorescence staining revealed that HCEP cells were holoclonal and expressed epithelial stem cell marker p63 without corneal cytokeratin 3. Cell viability remained unchanged after cells were cultured with 0.004, 0.04, and 0.4% TH in the medium, but it was significantly reduced when the concentration was increased to 3.33%. Cell migration, tested using scratch migration assay, was significantly enhanced when cells were cultured with TH at 0.04% and 0.4%. We also found that TH has hydrogen peroxide (H_2_O_2_) scavenging ability, although a trace level of H_2_O_2_ was detected in the honey in its native form. Preconditioning HCEP cells with 0.4% TH for 48 h showed better survival following H_2_O_2_-induced oxidative stress at 50 µM than untreated group, with a significantly lower number of dead cells (15.3±0.4%) were observed compared to the untreated population (20.5±0.9%, p<0.01). Both TH and ascorbic acid improved HCEP viability following induction of 100 µM H_2_O_2_, but the benefit was greater with TH treatment than with ascorbic acid. However, no significant advantage was demonstrated using 5-hydroxymethyl-2-furancarboxaldehyde, a compound that was found abundant in TH using GC/MS analysis. This suggests that the cellular anti-oxidative capacity in HCEP cells was augmented by native TH and was attributed to its antioxidant properties. In conclusion, TH possesses antioxidant properties and can improve cell migration and cellular resistance to oxidative stress in HCEP cells *in vitro*.

## Introduction

The cornea, which is a transparent and tough tissue that covers the anterior segment of the eye, is responsible for transmitting and reflecting light onto the retina. As the first layer of defence against external insults and oxidative insult from the sunlight, the corneal epithelium possesses mechanisms to maintain its cellular homeostasis. These mechanisms are orchestrated by a population of self-renewing stem cells that reside in the basal limbal epithelium, which is a niche area situated at the peripheral edge of the cornea called the limbus [1]. These cells express keratinocyte stem cell markers (p63, EGFR, K19, ABCG2 and integrin β1) and exhibit low expression of corneal differentiation markers (K3, involucrin, and connexin-43) [2], and they are responsible for sustaining the clarity and integrity of the corneal epithelium required for normal vision. Complete depletion or dysfunction of the corneal epithelial stem cells that occurs in some severe ocular surface abnormalities as a result of caustic injuries such as burns, inflammatory conditions, or hereditary genetic disorders such as aniridia [3], [4] will lead to conjunctivalisation, keratinisation, and opacification and result in impaired vision or blindness. Under these circumstances, visual acuity can only be restored by transplanting corneal epithelial stem cells onto the injured cornea. To minimise the risk of immune rejection, autologous stem cell transplantation may be the only option. Hence, culturing corneal epithelial stem cells and preserving their stemness and functions *in vitro* are pivotal for ensuring successful regeneration following transplantation.

Reactive oxygen species (ROS) are common metabolic by-products of aerobic metabolism, and their level is maintained through intrinsic antioxidant mechanisms in healthy cells. When maintained at the appropriate physiological level, ROS are vital in modulating several cellular signalling pathways that affect cell growth and function, including the phosphoinositide 3-kinase (PI3K) [5] and mitogen-activated protein kinase (MAPK) pathways [6]. In addition, ROS have the capability to dictate stem cell fate at physiological levels [7]–[9]. However, abnormal redox homeostasis involving ROS overproduction can induce oxidative stress, a physiological condition that renders cells susceptible to damage. Studies have confirmed that overproduction of ROS can compromise genomic stability [10] and trigger mutations and promote cancer growth [11]. High ROS levels also contribute to poor cell engraftment and viability, which impede regeneration after transplantation [12]. Although stem cells have greater antioxidant capacity compared to differentiated cells [13], [14], they can exhibit telomere shortening-induced replicative senescence and reduced self-renewal capability under oxidative stress [15]. Hence, protecting stem cells from oxidative damage may help to promote cell survival, homing, and regeneration after transplantation. This protection could be achieved by maintaining a reduced environment at the site of transplantation through adjunctive therapy with dietary antioxidant [16] or by adding an antioxidant supplement to cells during *in vitro* expansion prior to transplantation. The efficacy of the latter strategy is supported by studies showing the potential for supplemental antioxidant in the culture medium to enhance the intracellular antioxidant activity of stem cells [15], prevent cellular damage, and salvage culture-induced loss of stemness [17].

Tualang honey is a medicinal honey that is collected from the honeycomb of *Apis dorsata*, the bees that built their hives on the Tualang tree (*Koompassia excels*) that is common in tropical forests in Malaysia. This honey has been extensively studied and has been found to have therapeutic benefits in treating various medical conditions, including protecting post-menopausal bone structure [18], promoting burn wound contraction with antibacterial effects [19], [20], enhancing post-tonsillectomy healing [21], and inhibiting the growth of various cancer cells [22]–[24]. Generally, honey has been traditionally used to treat eye-related diseases [25], [26]. Uwaydat et al. (2011) recently showed that raw honey accelerated healing of corneal abrasions and attenuated the inflammatory response and angiogenesis in endotoxin-induced keratitis [27]. Similar to other types of honey, previous studies of Tualang honey have also described its anti-inflammatory and antioxidant properties in treating alkali-induced eye injury *in vivo* [28] and its ability to save keratinocytes from inflammation and DNA damage as a result of ultraviolet radiation *in vitro* [29].

Although many *in vivo* studies of the effects of Tualang honey have been conducted, the potential for using Tualang honey in the cultivation of stem cells has not been investigated. To date, only one study described the use of Tualang honey to supplement the culture medium when cultivating a human osteoblast cell line (CRL1543) [30]. Although many studies have shown the therapeutic benefits of Tualang honey in treating cornea injury [28], [29], its effects on corneal epithelial stem cells have yet to be evaluated. Herein we characterised the effects of Tualang honey on cytotoxicity, gene expression, and migration of human corneal epithelial progenitor (HCEP) cells and assessed its potential for improving cell resistance to oxidative stress.

## Methodology

### HCEP cell culture and expansion

HCEP cells were purchased from Gibco (Invitrogen Life Technologies Co., Carlsbad, CA, US) and ATCC (Manassas, VA, US). Cells were expanded in standard keratinocyte serum-free medium (KSFM, Gibco) that was supplemented with 5 ng/ml recombinant epidermal growth factor (rEGF) and 50 µg/ml bovine pituitary tissue extracts (Invitrogen Life Technologies Co., Carlsbad, CA, US). Passage 2–5 HCEP cells were used in all of the experiments.

### Preparation of Tualang honey

Tualang honey used in this experiment was from Federal Agriculture Marketing Authorities of Malaysia (FAMA) and was a gift from Professor Siti Amrah Sulaiman, Universiti Sains Malaysia. Tualang honey was diluted to 20% in serum-free DMEM/F12 (Gibco, Invitrogen Life Technologies Co., Carlsbad, CA, US) and filtered through a 0.2 µm syringe filter (Pall Co., Port Washington, NY, US) prior to use in cell culture. Filtered Tualang honey was further diluted in KSFM according to the dilution factor described in Table 1.

**Table 1:** Dilution factor for Tualang honey supplemented medium.

Tualang honey concentrations (%)	20% Tualang honey: KSFM
0.004%	1∶5000
0.04%	1∶500
0.4%	1∶50
3.33%	1∶5

Abbreviation: KSFM, keratinocyte-serum free medium.

### Immunofluorescence labelling

HCEP cells were seeded onto Lab-Tek8 well-chamber slides (ThermoSci., Logan, UT, USA) using honey supplemented medium. Cells were fixed with 4% paraformaldehyde at 4°C for 10 min and then permeabilised with phosphate buffered saline containing 0.1% Triton-X and 0.1% Tween-20 (all from Sigma, St Louis, MO, USA). Cell samples were washed with PBS and blocked with 10% goat serum (Cedarlane Lab, Ontario, Canada) for 1 h. The cells then were incubated with rabbit anti-p63 (Santa Cruz Biotechnology, Heidelberg, Germany) and mouse anti-keratin 3 (Clone AE5, Millipore, Billerica, MA, USA) primary antibodies overnight at 4°C. Samples were washed thrice with PBS and incubated with AlexaFluor488 goat anti-rabbit secondary antibody at 37°C for 2 h. Samples were then counterstained with DAPI and imaged using a confocal microscope (Olympus, Japan).

### RNA extraction and cDNA synthesis

The total RNA was isolated using the RNeasy Mini Kit (Qiagen, Hilden, Germany) according to the manufacturer's instructions. The isolated RNA was treated with DNase I (Sigma, St. Louis, MO, USA) to ensure the absence of genomic DNA contamination. Total RNA samples were quantitatively and qualitatively assessed using UV-spectrophotometric measurement and agarose gel electrophoresis, respectively. The cDNA was synthesised using the QuantiTect Reverse Transcription Kit (Qiagen, Hilden, Germany) according to manufacturer's protocol.

### Primer design

Full-length exon spanning gene sequences were obtained from the NCBI GenBank Database (http://www.ncbi.nlm.nih.gov/genbank/). All primers were designed from the gene sequences using Primer-BLAST online software (NCBI, NIH, USA). All primers are listed in Table 2. The sequences of the primers were compared to the GenBank Database using BLAST in order to determine their specificity. Primers that could have resulted in non-specific signals were excluded. All primers were purchased from BioBasic Inc. (Ontario, Canada).

**Table 2:** Sequences of primers used in this study.

Primers	Sequence (5′ – 3′)	Accession Number	PCR product (bp)
Beta-actin-F	GAGGCGTACAGGGATAGCA	NM_001101.3	302
Beta-actin-R	GTGGGCATGGGTCAGAAG		
ABCG2-F	GAGCTCGTCCCCTGGATGT	NM_004827.2	186
ABCG2-R	CGGAACCTTTTGAGTGGGCA		
Connexin43-F	CAAAATCGAATGGGGCAGGC	NM_000165.3	136
Connexin43-R	GCTGGTCCACAATGGCTAGT		
K12-F	CTCGCAGAGTGTGATAGGCA	NM_000223.3	146
K12-R	CCCCAAAGCCGGAACTAGAA		

Abbreviations: F, forward primer; R, reverse primer; PCR, polymerase chain reaction.

### Polymerase chain reactions (PCR)

PCR amplification was performed using the MyCycler Thermal Cycler (Bio-Rad, Hercules, CA, USA). All PCR reagents used in this study were purchased from Biotools B&M Labs (S.A., Madrid, Spain). Total cDNA used in each reaction was 1 µL unless specified otherwise. Reaction buffer (1 X), 1.5 mM MgCl_2_, 1.75 mM of each dNTP, 0.5 µM of each primer, 100 ng of cDNA, and 1 U of DNA Taq Polymerase were added to all samples to reach a final volume of 20 µl in each reaction. A negative control (sample without template) was also included. PCR was performed at 95°C for 2 min as initial denaturation followed by 35 cycles of 30 sec at 95°C, 30 sec at 60°C, and 30 sec at 72°C, and then at 72°C for another 2 min. The PCR products were loaded with gel loading dye (Promega, Madison, WI, USA) onto 4% agarose gel in 1X TAE buffer and electrophoresed for 60 min at 80 V. A 25 base pair molecular ladder (Promega, Madison, WI, USA) was used. Subsequently, the gel was stained with ethidium bromide (Bio-Rad, Hercules, CA, USA) to visualize the presence of PCR amplicons.

### Cytotoxicity assay

HCEP cells were seeded at 5000 cells/well onto a 96-well plate and left in an incubator with 5% CO_2_ at 37°C overnight. The culture medium then was changed to test medium supplemented with Tualang honey at various concentrations (Table 1), and the cells were incubated for 48 h. Cell viability was tested using AlamarBlue (Molecular Probes, Invitrogen Life Technologies Co., Carlsbad, CA, USA) according to the manufacturer's protocol. After 3 h of incubation with AlamarBlue, the fluorescence intensity of each sample was read using a FLUOstar Omega multi-mode microplate reader (BMG Labtech, Germany) with an excitation wavelength of 570 nm and an emission wavelength of 585 nm. Controls were HCEP cells in regular medium without Tualang honey. Cell viability was expressed as a percentage using the following formula: 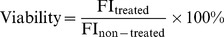



Where

FI_treated_ =  Fluorescence intensity of test samples (Tualang honey treated HCEP cells)

FI_non-treated_ =  Fluorescence intensity of control samples (non- treated HCEP cells)

### Scratch migration assay

HCEP cells were seeded onto a 6-well plate and allowed to expand and reach 90% confluence. A scratch was made onto each HCEP cell-containing well using a 200 µl pipette tip. The culture medium then was changed to standard KSFM supplemented with 0.004, 0.04, 0.4, or 3.33% (v/v) Tualang honey, and the cells were incubated at 37°C for 2 days. Ten representative images were taken using a charge-coupled device camera under an inverted light microscope pre- and post-treatment. Images obtained were analysed for wound closure using ImageJ software (NIH). All experimental samples were compared to a control (i.e., HCEP cells without Tualang honey treatment). The percentage of closure of the gap area was calculated using the following formula:
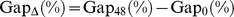



Where

Gap_Δ_ =  Occupied gap area after 48 h

Gap_48_ =  Gap area at 48 h

Gap_0_ =  Gap area at baseline

### Hydrogen peroxide inhibition assay

Tualang honey was prepared at 0.04, 0.4, and 4% and added to wells containing 5, 10, 20, 30, and 40 µM hydrogen peroxide (H_2_O_2_). The concentration of H_2_O_2_ was assessed using the Amplex Red hydrogen peroxide assay kit (Molecular Probes, Invitrogen Life Technologies Co., Carlsbad, CA, USA) after 30 min of incubation at room temperature according to the manufacturer's protocol. The excitation and emission wavelengths were set at 544 and 590 nm, respectively, to obtain the fluorescence reading using FLUOstar Omega multi-mode microplate reader.

### Oxidative stress assay

HCEP cells were cultured with 0.004, 0.04, 0.4, and 3.33% (v/v) Tualang honey in supplemented KSFM for 48 h. Treated cells then were exposed to 50 µM H_2_O_2_ for 24 h. The number of dead cells in treated groups after H_2_O_2_ exposure was measured using propidium iodide staining (BD Bioscience Franklin Lakes, NJ, USA) and quantified using a flow cytometer (FACS Canto, BD Bioscience Franklin Lakes, NJ, USA). Untreated HCEP cells served as the negative control. Ascorbic acid, a potent anti-H2O2 agent used as a positive control and 5-hydroxymethyl-2-furancarboxaldehyde were both purchased from Sigma, St Louis, MO, USA.

### Gas Chromatography-Mass Spectrometry (GC-MS) analysis

GC-MS analysis of the honey was conducted using a Shimadzu system (Japan) consisting of a GC-MS-QP2010 gas chromatograph and a quadrupole mass spectrometer. The interface and source temperatures were 280°C and 250°C, respectively. Electron impact mass spectra were recorded in the 20–650 amu range at 70 eV ionization energy. Separation was performed on a fused-silica bonded-phase capillary column BP X5 (30 mx0.25 mm ID and 0.25 µm film thickness). The injector temperature was set at 250°C; and the samples were run in split mode with the ratio being adjusted to 25∶1 and an injection volume of 1 µL. The temperature program was isothermal at 35°C for 1 min, then it was raised to 280°C at 25°C/min and to 310°C at 10°C/min. This temperature was held for 2 min. Samples in the chromatograms were identified by comparing their mass spectra with NIST08 library data as well as the retention times against known standards.

### Statistical analysis

All experiments were repeated three times and data were expressed as mean ± standard error (SEM). All statistical analyses were performed using Graphpad Prism (La Jolla, CA, USA). The differences between groups were analysed using one-way ANOVA with Dunnett post-hoc test. Differences were considered significant at *p*<0.05.

## Results

### Cultured HCEP cells possessed stem cell characteristics

**Figure 1:**
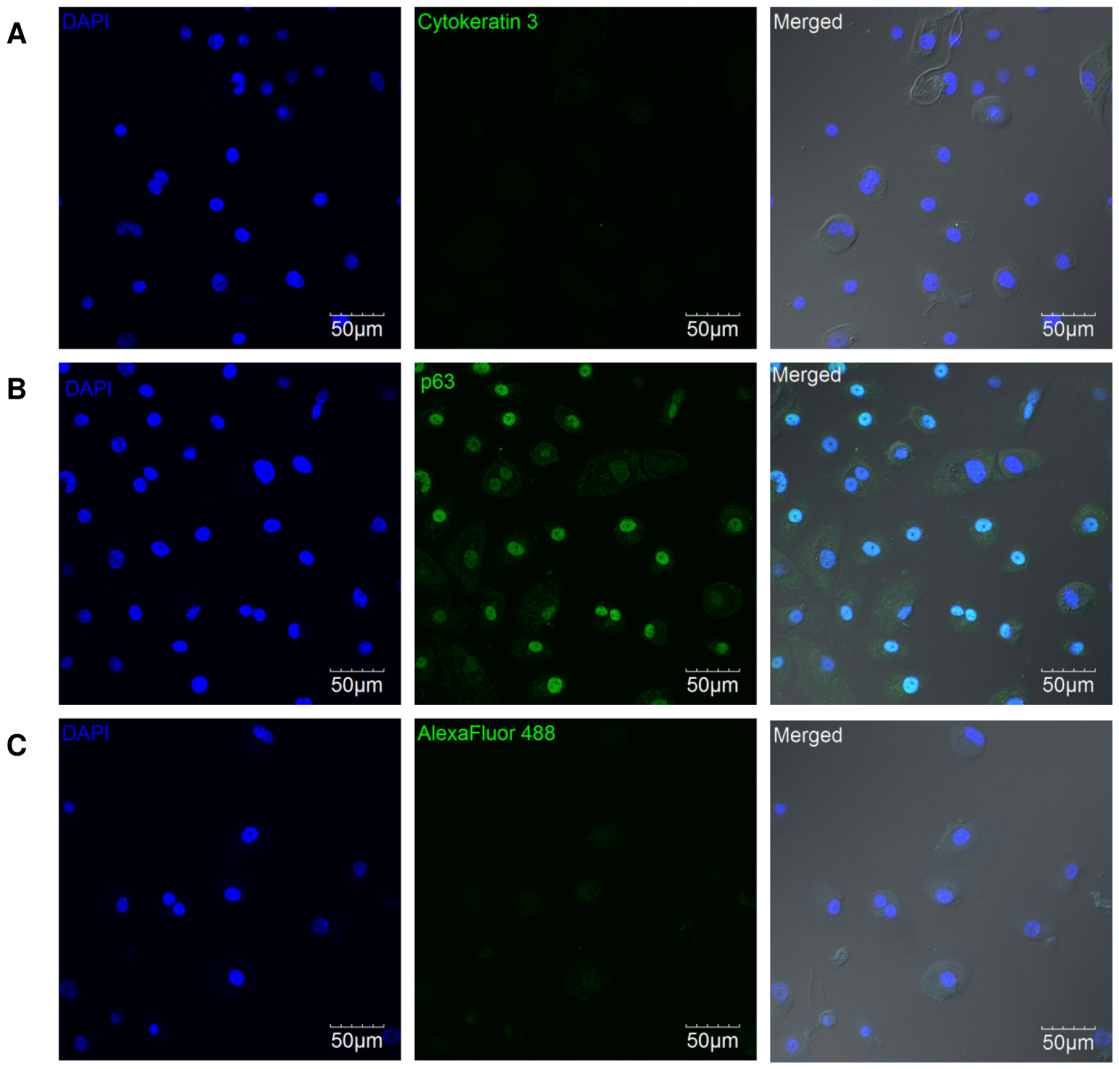
Representative confocal images of immunofluorescence-labelled HCEP cells. HCEP cells did not express the cornea-specific marker cytokeratin 3 (green) (A) but did express nuclear p63 transcription factor (green) (B). HCEP cells stained with AlexaFluor488 secondary antibody without any primary antibody were served as the negative control (C). Abbreviations: DAPI, 4′,6-diamidino-2-phenylindole.

The cultured HCEP cells were highly proliferative and capable of forming holoclones. Immunofluorescence staining confirmed that the cultured HCEP cells used in this study expressed nuclear p63 but not cytokeratin 3/12 (Figure 1), suggesting a keratinocyte stem cell phenotype. However, the cultured HCEP cells exhibited signs of replicative senescence as soon as they reached passage 5 or 6.

### Low levels of Tualang honey favoured HCEP cell viability, gene expression, and migration

**Figure 2:**
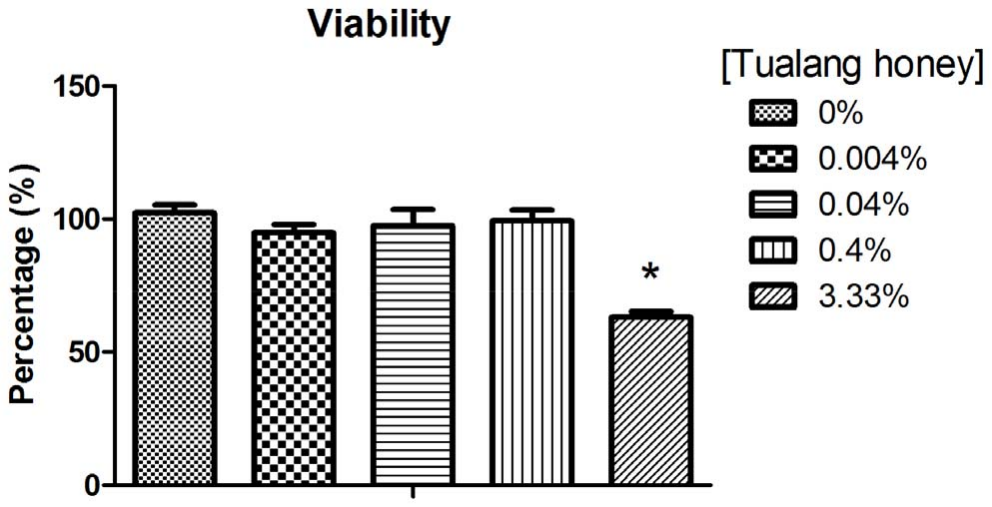
HCEP cell viability after treatment with 0, 0.004, 0.04, 0.4, and 3.33% Tualang honey for 48 h. Significant lower viability was observed in HCEP cells treated with 3.33% Tualang honey compared to the other treatments. ***p<0.001.

**Figure 3:**
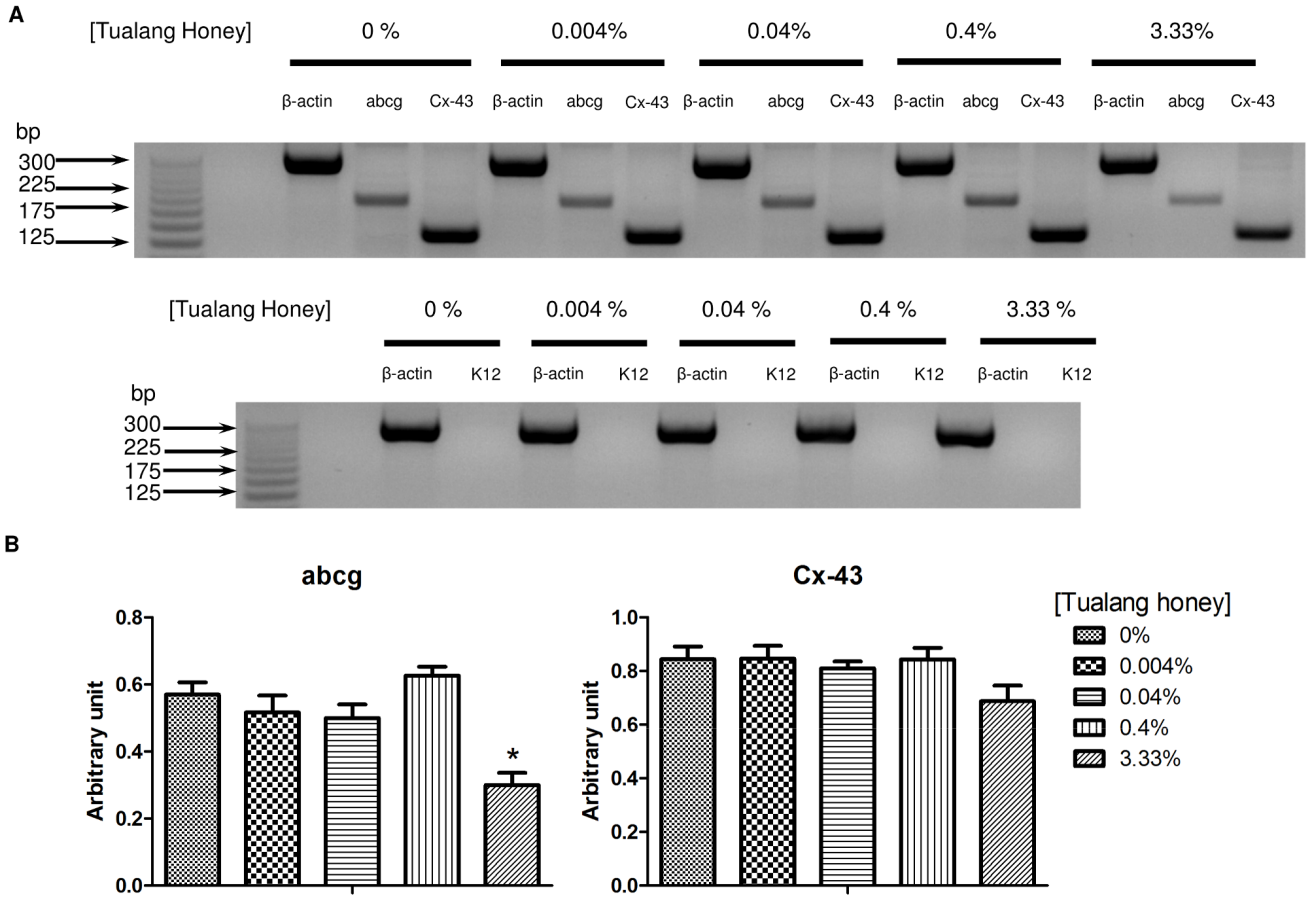
mRNA expression of Tualang honey treated HCEP cells. (A) RTPCR showed abcg and connexion-43 mRNA expressions (normalised to β-actin expression) but not cytokeratin-12 expression in HCEP cells. (B) abcg mRNA expression was down-regulated in HCEP cells treated with 3.33% Tualang honey, but the mRNA for connexin-43 remained unchanged in all groups. *p<0.05 compared to untreated control (0%). Abbreviations: abcg, ATP-binding cassette transporter; Cx-43, connexion-43.

Viability of HCEP cells was assessed after 24 h and no sign of cytotoxicity was observed in groups treated with 0.004, 0.04, or 0.4%Tualang honey (Figure 2). However, cell viability was significantly reduced to 63.1±2.1% at the concentration of 3.33% compared to the untreated control (*p*<0.001). We also sought to investigate whether Tualang honey could affect HCEP cell stemness or trigger corneal epithelial differentiation. To do this, the mRNA level of ATP-binding cassette transporter 2 (abcg2), a marker which was found to express in human corneal epithelial stem cells, and corneal epithelial differentiation marker connexin-43 and cytokeratin-12, were examined. RTPCR revealed that the mRNA expression for connexin-43 and abcg2 were detected in all groups, but expression of cytokeratin-12 was not (Figure 3A). Semi-quantitative analysis of the expressed mRNA revealed that connexin-43 mRNA remained unaffected by Tualang honey in all groups, which also indicates that Tualang honey had minimal or no potential in triggering corneal epithelial differentiation. However, a significant down-regulation of abcg2 mRNA was found in HCEP cells treated with 3.33% Tualang honey; this represents a decrease of 47% when compared with the expression in untreated cells (*p*<0.05) (Figure 3B). These results suggest that Tualang honey did not affect the HCEP stemness and differentiation at non-cytotoxic level.

**Figure 4:**
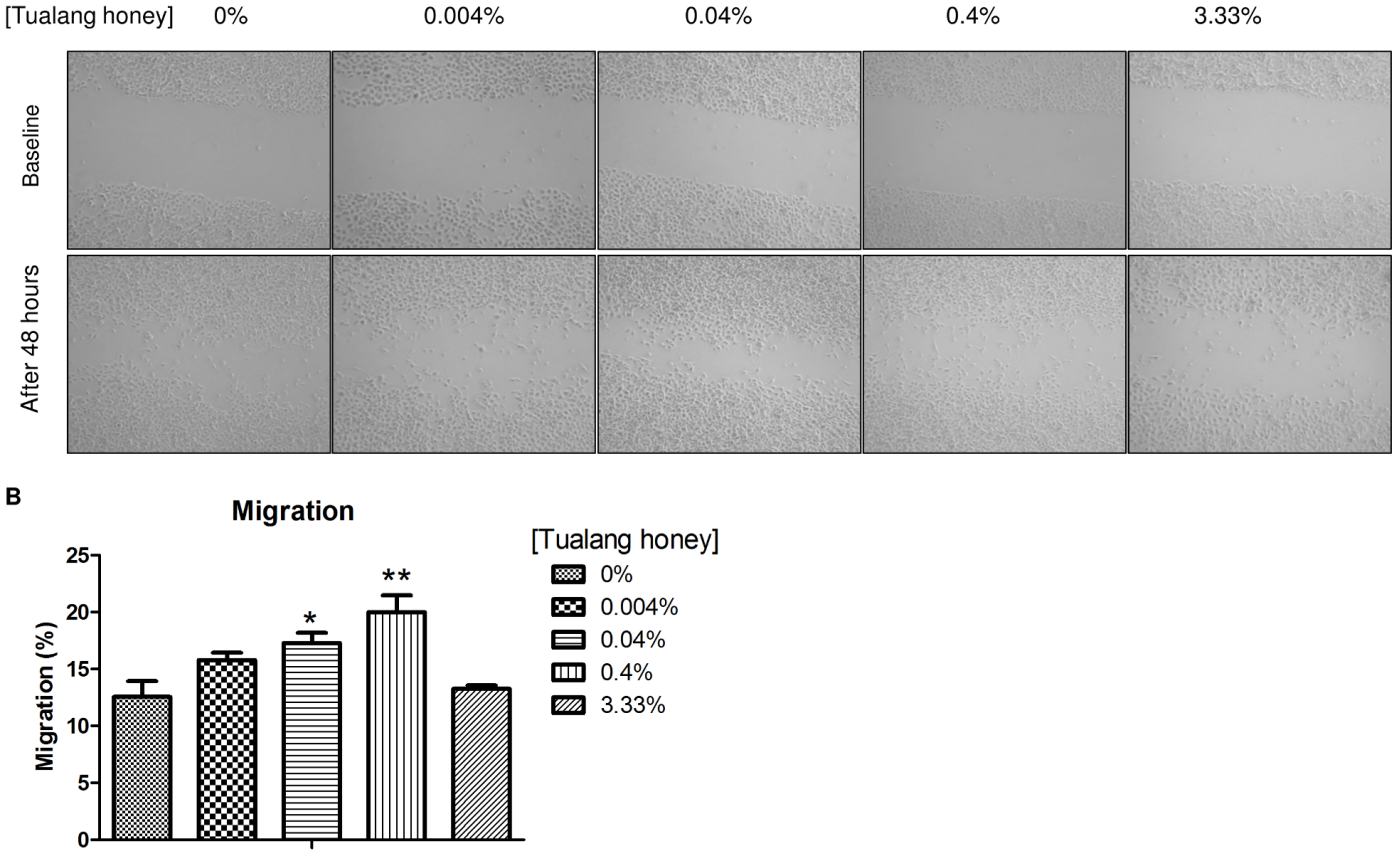
HCEP cell migration following treatment with Tualang honey. (A) Representative images of the HCEP cell scratch migration assay at baseline and after 48 h. (B) The gap size occupied by HCEP cells after 48 h was largest in the 0.4% Tualang honey treatment (*p<0.05; **p<0.01 compared to untreated control).

The *in vitro* scratch migration assay showed that the percentage of gap area covered by migrated HCEP cells after 48 h of treatment increased in a dose-dependent manner (Figure 4). HCEP cells treated with 0.04 and 0.4% Tualang honey occupied 17.3±0.9% (*p*<0.05) and 20±1.5% (*p*<0.01) of the gap area, respectively, compared to 12±6% in the untreated group. In the 3.33% Tualang honey treatment, only 13.3±0.3% of the gap area was occupied after 48 h.

### Tualang honey scavenged H_2_O_2_ in a dose-dependent manner and enhanced HCEP cell resistance to oxidative stress

**Figure 5:**
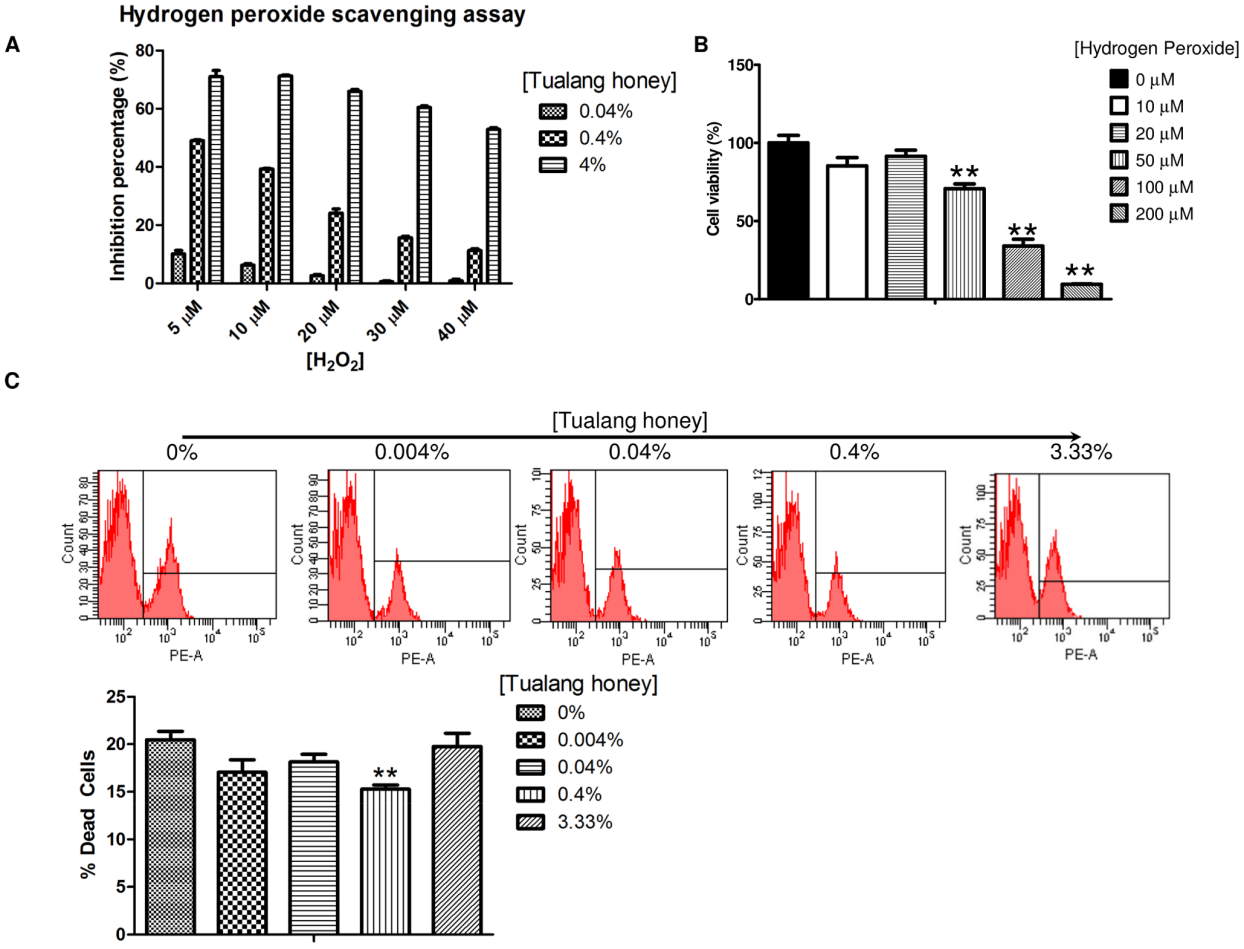
Effects of Tualang honey on hydrogen peroxide and HCEP cell resistance to oxidative stress. (A) *In vitro* inhibitory effects of Tualang honey at 0.04, 0.4, and 4% (v/v) against 5, 10, 20, 30, and 40 µM H_2_O_2_. (B) HCEP viability in response to H_2_O_2_ induction at 0, 10, 20, 50, 100 and 200 µM after 24 hours using AlamarBlue assay. (C) Flow cytometric analysis showed the total number of PI-stained dead cells in Tualang honey treated HCEP cells in response to exposure to 50 µM H_2_O_2_ after 24 h (**p<0.01 compared to untreated control). Abbreviation: PI, propidium iodide.

When Tualang honey was tested for its H_2_O_2_ scavenging ability, the level of inhibition was found to increase with increasing Tualang honey concentration (Figure 5A). This result suggests that Tualang honey is a source of anti-H_2_O_2_ antioxidant. Notably, only negligible scavenging effects were observed at 30 and 40 µM H_2_O_2_ when the Tualang honey was diluted to 0.04%. This indicates that over-dilution of Tualang honey may compromise its H_2_O_2_ scavenging ability. To identify the cytotoxicity of H_2_O_2_, HCEPs were exposed to 10, 20, 50, 100 and 200 H_2_O_2_ and the viability was assessed after 24 h using AlamarBlue assay. As shown in Figure 5B, HCEP viability was reduced by H_2_O_2_ in a dose-dependent manner, and the difference became apparent and statistically significant when H_2_O_2_ level was increased to ≥50 µM (*p*<0.01).

This study was extended to investigate the potential for Tualang honey to improve HCEP cell resistance to H_2_O_2_-induced oxidative stress. Interestingly, HCEP cells treated with 0.4% honey had a higher survival rate than the untreated HCEP cells, with a significantly lower number of dead cells (15.3±0.4%) compared to the control (20.5±0.9%, p<0.01) (Figure 5C).

### Tualang honey contains active compounds with known antioxidant properties

**Figure 6:**
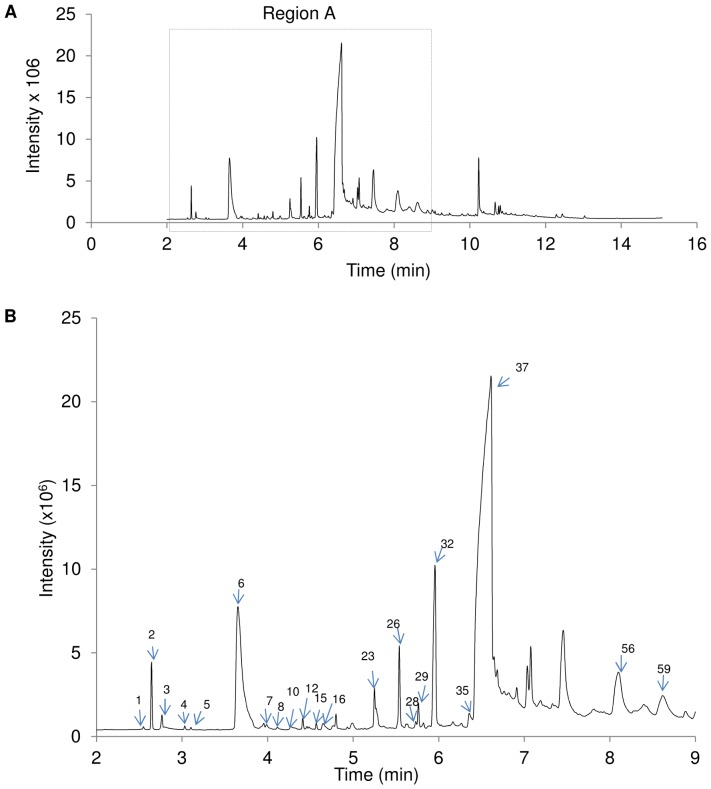
GC-MS analysis of Tualang honey. (A) Region A in the chromatogram of 20% Tualang honey diluted in distilled water. (B) Region A was enlarged and analysed further to identify the phytochemical constituents in Tualang Honey based on the NIST08 Mass Spectral Library.

The chromatogram of filtered 20% Tualang honey diluted in distilled water revealed the presence of several volatile compounds (Figure 6). Of those, only 20 chemical constituents were identified (Table 3). The major constituent in Tualang honey was 5-hydroxymethyl-2-furancarboxaldehyde (5HMF), a five carbon ring aromatic aldehyde antioxidant that accounted for a relative peak area of 36.21% [31]. Other known antioxidants, such as 3-furaldehyde (7.46%), phenylacetaldehyde (1.13%), 2-furanmethanol (0.13%), and maltol (0.12%), were also present in Tualang honey [31], [32], albeit at lower percentages.

**Table 3:** Phytochemical constituents detected in Tualang honey.

Peak	Area (%)	Phytochemical	Activity
37	36.21	5-(hydroxymethyl) 2-Furancarboxaldehyde,	Antioxidant [31]
6	7.46	3-Furaldehyde	Antioxidant [32]
56	4.73	Beta.-D-Glucopyranose, 1,6-anhydro-	-
32	4.23	4H-Pyran-4-one, 2,3-dihydro-3,5-dihydroxy-6-methyl-	Antimicrobial, anti-inflammatory [60], [61]; antioxidant [62]–[64]
59	3.06	1,6-Anhydro-.beta.-D-glucofuranose	-
26	1.18	Methyl 2-furoate	-
23	1.13	Phenylacetaldehyde	Antioxidant [65]
2	0.71	Formic acid	-
29	0.33	Levoglucosenone	Anticancer, treatment for autoimmune system and cardiovascular diseases [66]
16	0.2	2-Furancarboxaldehyde, 5-methyl-	-
3	0.16	Acetic acid	Antihistamine [67]
12	0.14	2(5H)-Furanone	-
7	0.13	2-Furanmethanol	Antioxidant [68]–[70]
28	0.12	Maltol	Antioxidant [71], [72], anticonvulsant, depressant [73], anti-aging [74]
35	0.1	2(3H)-Furanone, dihydro-4-hydroxy-	-
8	0.09	Propanoic acid, 2-hydroxy-, ethyl ester	-
15	0.09	2(5H)-Furanone, 5-methyl-	-
10	0.08	2-Propanone, 1,3-dihydroxy-	-
4	0.04	2-Propanone, 1-hydroxy- (CAS) Acetol	-
1	0.03	Hydrogen chloride	-
5	0.02	Propanoic acid, 2-oxo- (CAS) Pyruvic acid	-

### The improvement in HCEP resistance to H_2_O_2-_induced oxidative stress was attributed to the antioxidant properties of Tualang honey at its native form

**Figure 7:**
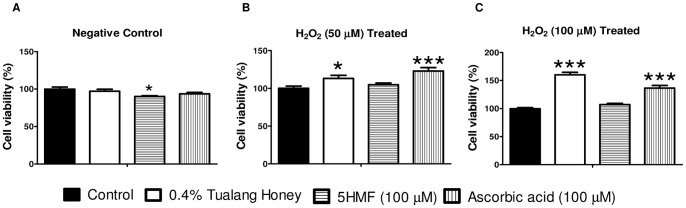
AlamarBlue cell viability assay of Tualang honey (0.4%), ascorbic acid (100 µM) and 5HMF(100 µM) treated HCEP cells after H_2_O_2_ induction. The resistance of treated cells against H_2_O_2_ was tested at (A) 0, (B) 50 and (C) 100 µM for 24 h. Significant difference in viability was found between Tualang honey and ascorbic acid-treated group using ANOVA with Tukey multiple comparisons test (*p<0.05; ***p<0.01 compared to untreated control). Abbreviation: 5HMF, 5-hydroxymethyl-2-furancarboxaldehyde.

To test whether the observed improvement in HCEP resistance to H_2_O_2_-induced oxidative stress following the treatment with Tualang honey was attributed to 5-hydroxymethyl-2-furancarboxaldehyde (5HMF), the viability of HCEPs treated with 100 µM of 5HMF for 48 h were examined with AlamarBlue after exposed to 50 and 100 µM H_2_O_2_ for 24 h. As shown in Figure 7, HCEP viability was significantly decreased after treated with 5HMF for 48 h compared to the untreated group (*p*<0.05). However, the viability became comparable to that of untreated control after 24 h induction of H_2_O_2_ at 50 and 100 µM. This indicates that although 5HMF had little effects in improving HCEP resistance to H_2_O_2_, the benefit was nullified by its mild cytotoxicity.

To assess whether the antioxidant property of Tualang honey was responsible for the elevated resistance to H_2_O_2_-induced oxidative stress, HCEPs treated with 100 µM ascorbic acid, a known powerful antioxidant, was included as the positive control. AlamarBlue assay showed better HCEP survival following H_2_O_2_ insults at 50 µM and 100 µM after treated with ascorbic acid or Tualang honey as compared to the untreated group. More importantly, the resistance to 100 µM H_2_O_2_ was found significantly greater in Tualang honey-treated HCEP cells than that of ascorbic acid-treated (Figure 7C). Taken together, these results confirm that the enhanced HCEP resistance to oxidative stress is conferred by the antioxidant property of Tualang honey in its native form.

## Discussion

Tualang honey has been used to treat bacterial infections [33], promote burn wound healing [19], [34], and protect bone in post-menopausal women [18]. However, its potential as a supplement in cell culture medium for stem cell expansion has not been studied, and little is known about the effects of honey on cells at the cellular level. This is the first study to evaluate the use of Tualang honey in culturing HCEP cells and its effects in enhancing cell function *in vitro*. Thus, it is a pivotal study for exploring the potential of this honey and its application in corneal regeneration.

The ATP binding cassette transport 2 (abcg2) is known for its capability to efflux Hoechst 33342 and has been used as a marker to identify side population cells [35]. This marker was also found to present in clonogenic human limbal-derived epithelial stem cells [36]. A study by Kubota et al. (2010) suggested that abcg2 has a vital role in maintaining endogenous anti-oxidative capacity in HCEPs [37]. Here, the cultured human corneal epithelial cells used in this study expressed mRNA for abcg2 and nuclear p63 protein, the putative corneal epithelial stem cell markers [38], [39]. However, significant downregulation of abcg2 mRNA was detected when Tualang honey was introduced to the cells at cytotoxic level, 3.33%. This may also suggest that the cytotoxicity of Tualang honey in HCEP cells is via the downregulation of abcg2 expression, reduces the cell anti-oxidative capacity which renders the cells susceptible to oxidative damage. Although no terminal corneal epithelial differentiation was detected (based on the lack of cytokeratin 3 protein and cytokeratin 12 mRNA expression), we found that the cells constantly expressed gap junction protein connexin-43 mRNA, a negative marker for corneal epithelial stem cells [40]. This suggests that the cells had partial commitment to the corneal epithelial lineage. Hence, in order to accurately describe the cell population, we called them human corneal epithelial progenitor cells (i.e., HCEPs).

Previous studies showed that the bactericidal effects of honey were partly, if not entirely, due to its acidity, high osmolality, and H_2_O_2_ content [41], [42]. These characteristics could also be cytotoxic to cells. We addressed the cytotoxicity of Tualang honey to cultured HCEP cells by lowering the concentration to a level that favoured cell growth (≤0.4%). Our data show that the pH of the culture medium was not altered by Tualang honey at low concentration (0.004–0.4%), but the medium became acidic at high concentration (3.33%) (Table 4). Furthermore, a high level of honey supplementation could produce a hyperosmotic culture medium, which could induce ROS generation and apoptosis in corneal epithelial cells [43]. In contrast to our finding, Ghashm *et al.* (2010) reported that the pH of the culture medium remained suitable for cell culture after adding Tualang honey at high concentrations (3.5–20%) compared to the control medium without Tualang honey [23]. This discrepancy may be due to differences in preparation and storage conditions of the Tualang honey used in the experiments, as storage conditions can alter the content and properties of the honey [44].

**Table 4:** pH values of HCEP cell culture medium after supplementation with Tualang honey at various concentrations.

Tualang Honey concentrations	0%	0.004%	0.04%	0.4%	3.33%	5.0%	10.0%
pH	7.65	7.67	7.58	7.59	7.17	5.83	4.81

We also found that Tualang honey diluted to 0.04% did not affect gene expression and was favourable to HCEP cells, as shown by the enhanced cell migration *in vitro* in this treatment. This result suggests that dilution of Tualang honey does not hamper its beneficial effects on HCEP cells. These data coincide with a past study that showed improvement in proliferation of human osteoblast cells (CRL 1543) when the culture medium was supplemented with 0.0195% Tualang honey [30]. The underlying mechanism for the observed improvements is unknown, but it could be related to the active compounds present in Tualang honey or to the presence of H_2_O_2_, a ROS that is essential for modulating stem cell behaviours at the physiological level [45]–[47]. Pan *et al.* (2011) recently suggested that low levels of H_2_O_2_ (10–50 µM) could promote rabbit corneal epithelial cell attachment, mobility, and wound repair [48]. This contradicts our findings using human cells that H_2_O_2_ showed significant cytotoxicity at 50 µM and no noticeable changes in cell number at 10 and 20 µM when compared with the untreated control. Nonetheless, this discrepancy may due to the difference in the species of origin of corneal epithelial cells used in the experiments.

Furthermore, we also found that H_2_O_2_ was present in Tualang honey, but only at a negligible level of <2 µM after dilution to ≤0.4%. This finding suggests that the H_2_O_2_ level may not be sufficient to exert significant changes in cell behaviours. Although H_2_O_2_ is present in naturally occurring Tualang honey, it also contains several phytochemical compounds with antioxidant properties that are capable of mitigating H_2_O_2_. The predominant phytocompound identified in Tualang honey was 5-hydroxymethyl-2-furancarboxaldehyde, which is a five carbon ring aromatic aldehyde antioxidant that is commonly found in fruits [49] and marine products [50]. Compared to other types of honey, Tualang honey had been identified as a new source of antioxidant with superior activity that is attributed to its high phenolic content [51].

Oxidative stress has been shown to cause chromosomal instability, shortened telomeres, and cellular replicative senescence in stem cells *in vitro* [52], [53], all of which hamper cell function and limit the therapeutic outcome of cell transplantation. Accumulating evidence supports the premise that cellular antioxidant levels in stem cells play a vital role in dictating their fate, regenerative capability, and therapeutic outcome after transplantation [54], [55]. Overexpression of cellular redox regulators such as Cu/Zn superoxide dismutase enzymes and nuclear factor erythroid 2-related factor-2 (Nrf-2) has been shown to improve stem cell functions [56]. The oxidative stress tolerance of stem cells can also be augmented by the addition of exogenous catalase [57] or antioxidants from natural products [16], [58], [59]. Herein, Tualang honey at 0.4% was found to improve HCEP cell resistance to oxidative stress, as shown by the significantly lower number of dead cells after treatment with 50 µM H_2_O_2_ in the treatment versus control group. This indicates that Tualang honey can be used as a source of antioxidant for preconditioning HCEP cells prior to transplantation. However, more evidence is needed to confirm the changes in HCEP cell antioxidant enzyme levels that likely account for the Tualang honey-induced enhancement of the oxidative stress tolerance of HCEP cells.

In summary, this study describes a novel approach to integrating the application of natural products to the process of corneal regeneration. Our data indicate that Tualang honey is an antioxidant and contains active phytocompounds that enhance HCEP cell migration and resistance to oxidative stress *in vitro* in a dose-dependent manner. The beneficial effects, however, are offset by the cytotoxicity of Tualang honey at high concentration. In-depth studies to isolate and identify the active components in Tualang honey and to identify the mechanism responsible for the observed benefits *in vitro* and *in vivo* are needed to enable use of this natural product for the treatment of corneal diseases.
