# Correction: Tualang Honey Improves Human Corneal Epithelial Progenitor Cell Migration and Cellular Resistance to Oxidative Stress *In Vitro*

**DOI:** 10.1371/journal.pone.0105233

**Published:** 2014-08-01

**Authors:** The PLOS ONE Staff

The name of the university is missing from the affiliation for co-author, Dr. Yoke Keong Yong. Please refer to the correct affiliation below:

Department of Human Anatomy, Faculty of Medicine and Health Sciences, Universiti Putra Malaysia, Serdang, Selangor Darul Ehsan, Malaysia
